# Evaluation of the antimicrobial and cytotoxic potential of endophytic fungi extracts from mangrove plants *Rhizophora stylosa* and *R. mucronata*

**DOI:** 10.1038/s41598-022-06711-9

**Published:** 2022-02-17

**Authors:** Jing Zhou, Zhao Feng, Wenfang Zhang, Jing Xu

**Affiliations:** 1 One Health Institute, School of Chemical Engineering and Technology, Hainan University, Haikou, 570228 People’s Republic of China; 2 Hainan Provincial Fine Chemical Engineering Research Center, School of Life Sciences, Hainan University, Haikou, 570228 People’s Republic of China

**Keywords:** Biological techniques, Biotechnology, Drug discovery, Microbiology

## Abstract

Mangrove endophytic fungi are tolerant to numerous stresses and are inevitably capable of exhibiting excellent biological activity by producing impressive numbers of metabolites with special biological functions, based on previous work on the biological potential of mangrove-derived endophytic fungi. To obtain marked antimicrobial and cytotoxic fermentation products of culturable endophytic fungi from mangrove forests, our research evaluated the antimicrobial and cytotoxic activities of crude extracts of endophytic fungi from *Rhizophora stylosa* and *Rhizophora mucronata.* Forty-six fungal isolates were cultured on four different media, namely, dextrose agar (PDA), Czapek’s agar (CZA), rice medium (RM) and grain medium (GM) and harvested by ethyl acetate solvent at 40 days. The extracts were tested for antimicrobial activity by the microdilution method against the gram-negative bacteria *Pseudomonas adaceae* (PA), gram-positive bacteria *Enterococcus faecalis* (EF), methicillin-resistant *Staphylococcus aureus* (MRSA) and pathogenic fungus *Monilia albicans* (MA). The cytotoxic activity of the extracts was evaluated by MTT assay using A549 human lung cancer cells, HeLa human cervical carcinoma cells, and HepG2 human hepatocellular cells. The results showed that rice medium could promote the secretion of antimicrobial and antitumour secondary metabolites of endophytic fungi in comparison with other cultivation media. Seventeen strains (68%) from *R. stylosa* exhibited inhibitory effects on indicators, especially *N. protearum* HHL46, which could inhibit the growth of four microbes with MIC values reaching 0.0625 mg/mL. Fifteen strains (71.4%) from *R. mucronata* displayed activities against human pathogenic microbes; in particular, *Pestalotiopsis *sp*.* HQD6 and *N. protearum* HQD5 could resist the growth of four microbes with MIC values ranging from 0.015 to 1 mg/mL. In the cytotoxicity assay, the extracts of 10 strains (40%), 9 strains (40%) and 13 strains (52%) of *R. stylosa* and 13 strains (61.9%), 10 strains (47.6%) and 10 strains (47.6%) of *R. mucronata* displayed cytotoxicity against A549, HeLa and HepG2 cancer cells with cell viability values ≤ 50%. *Neopestalotiopsis protearum* HHL46, *Phomopsis longicolla* HHL50, *Botryosphaeria fusispora* HQD83, *Fusarium verticillioides* HQD48 and *Pestalotiopsis *sp*.* HQD6 displayed significant antitumour activity with IC_50_ values below 20 μg/mL. These results highlighted the antimicrobial and antitumour potential of endophytic fungi from *R. stylosa* and *R. mucronata* and the possibility of exploiting their antimicrobial and cytotoxic agents.

## Introduction

For decades, natural products have been among the most successful sources of drugs to treat infectious diseases, such as penicillin, vancomycin and daptomycin^1^. However, their indiscriminate use has led to a relentless and pernicious emergence of antimicrobial resistance to all major classes of these drugs and the emergence of new and reemergent infectious diseases^2,3^. As a consequence, there is an alarming scarcity of new antibiotic classes in the pipelines of the pharmaceutical industry. Cancer is the second leading cause of death worldwide, and the mortality of the common forms of cancer is unacceptably high^4^. Conventional therapies cause serious side effects and, at best, merely extend the patient’s lifespan by a few years. The mortality statistics are unlikely to change until there is a reorientation of concepts for the use of natural products as new chemopreventive agents^5^. Hence, naturally derived compounds have received increasing attention in the past 40 years for the discovery of novel cancer preventive and therapeutic agents. Over 60% of clinically useful anticancer agents and a considerable number of natural products or analogues derived therefrom are in clinical and preclinical development^6^. Examples include taxanes, Vinca alkaloids, podopyllotoxins and camptothecin^7^. The anticancer drug potential of microbes remains relatively unexplored, but it is becoming increasingly evident that the realm of microbes from unusual or specialized ecological niches, such as mangrove habitats, offers vast untapped potential.

Mangrove forests are complex ecosystems that are distributed in the intertidal zone of tropical and subtropical coasts, which not only refers to mangrove plants but also includes diverse groups of microorganisms formed by abundant biological communities^8^. There are 61 species of true mangrove plants in the world, belonging to 14 families and 21 genera. China has 26 species, and 24 of them have been documented in Hainan^9^. Mangrove endophytic fungi tolerant of numerous stresses, such as high salinity, high temperature, extreme tides, oxygen pressure, high humidity, and light and air limitations, have evolved unique metabolic pathways for the purposes of competition over nutrition and space in extremely fierce niches, which will inevitably render them capable of exhibiting excellent biological activity by producing impressive numbers of metabolites with special biological functions^10,11^. Rivai et al. studied the antimicrobial activities of endophytic fungi from *R. mucronata,* and ethyl acetate extracts of 14 strains (64.3%) showed activity against the test microbes *S. aureus*, *E. coli*, and *C. albicans*^12^. *Cladosporium* sp. isolated from *Thespesia populneoides* and *Xylaria *sp. isolated from *Acanthus ilicifolius* were reported to exhibit gram-positive and gram-negative bacterial inhibition towards *Bacillus subtilis*, *Pseudomonas aeruginosa*, *Escherichia coli* and *Staphylococcus aureus*. Additionally, most extracts of 84 endophytic fungi from 10 different mangrove hosts belonging to seven families were Rhizophoraceae (*Rhizophora apiculata*, *R. mucronata*, *Ceriops decandra*), Sonneratiaceae (*Sonneratia alba*), Combretaceae (*Lumnitzera littorea*), Avicenniaceae (*Avicennia alba*), Acanthaceae (*Acanthus ilicifolius*), Meliaceae (*Xylocarpus granatum* and *Xylocarpus moluccensis*) and Malvaceae (*Thespesia populneoides*) and showed cytotoxicity against some cancer cell lines, including A375 (human malignant melanoma), SW620 (human colorectal adenocarcinoma), Kato III (human gastric carcinoma), HepG2 (human liver hepatoblastoma) and Jurkat (human acute T cell leukaemia)^13^. Extracts of 9 endophytic fungal isolates (64.3%) from *R. mucronata* can inhibit the growth of the tested bacteria and fungi, of which 12 isolates (85.7%) were cytotoxic (cell viability < 50%) against T47D cells^12^.

*Rhizophora* is one of the most conspicuous genera of the most widespread mangrove family, Rhizophoraceae. Sixty-six endophytic fungi were isolated from plants of the genus *Rhizophora*, such as *Aspergillus*, *Cladosporium*, *Chaetomium*, *Fusarium*, *Lasiodiplodia*, *Penicillium*, *Pestalotiopsis*, *Phomopsis*, *Phoma*, *Phyllosticta*, and *Trichoderma*, more than 195 natural products, including alkaloids, terpenoids, coumarins, chromones, quinones, peptides, phenolic acids, and lactones, were identified, and cytotoxicity was found to be the most notable bioactivity of the secondary metabolites isolated^9^. Among them, unprecedented scaffolds indole-diterpenes rhizovarins A–C isolated from *Mucor irregularis* QEN-189 in *R.* stylosa demonstrated activity against human cancer HL-60 and A-549 cell lines with IC_50_ values ranging from 5 to 15 μM and are novel inhibitors of the Wnt/β-catenin pathway in breast cancer cells^14–16^. A novel cytochalasin, which was found in the endophytic fungus *Phomopsis *sp. derived from *Kandelia candel*, can effectively induce apoptosis and inhibit the migration of A549 human lung cancer cells by significantly increasing the protein expression of Bax, p53 and cleaved Caspase-3 and increasing the ratio of the antiapoptotic proteins Bax/Bcl-2^17,18^. In addition, demethylincisterol A_3_ was a selective inhibitor of the classical nonreceptor protein tyrosine phosphatase Shp2 and isolated from the endophytic *R. mucronata Pestalotiopsis *sp*.* HQD6^19,20^. The new polyketide derivative pestalpolyol I was obtained from extracts of the endophytic fungus *P. clavispora* isolated from *R. harrisonii* and exhibited strong cytotoxicity against the mouse lymphoma cell line L5178Y with an IC_50_ value of 4.10 μM^21^.

During our previous work on the biological potential of mangrove-derived endophytic fungi, we isolated and identified endophytic fungi from mangroves *R. stylosa and R. mucronata,* and their antioxidant activities were evaluated^22^. In this study, we continuously investigated the antimicrobial and cytotoxic activities of endophytic fungi isolated from *R. stylosa and R. mucronata*. We tried to promote the secretion of antibacterial and antitumour substances of our fungal isolates by using four different culture media. Four microbial indicator strains (PA, EF, MRSA and MA) and three human cancer cell lines (HeLa, A549 and HepG2) were adopted for antimicrobial and cytotoxicity tests, respectively. This study aims to provide constructive information on the in vitro potential of the endophytic fungi of these two hosts as producers of antimicrobial and cytotoxic activities.

## Results

### Antimicrobial activity of fungal extracts

From a total of 46 fungal extracts assayed, 32 extracts (69.6%) showed antimicrobial activity against at least one of the indicator pathogenic microbes tested (Tables 1 and 2). The antimicrobial activity of the same isolated fungal strain was significantly different (P < 0.05) when cultured on different media, but all of the antimicrobial activities were weaker than that of the positive control.

**Table 1:** Antimicrobial acticity of the endophytic fungi from *R. stylosa.*

Number	Species	MIC mg/mL															
		PDA^a^				CZA				RM				GM			
		M.A^b^	MRSA	P.A	E.F	M.A	MRSA	P.A	E.F	M.A	MRSA	P.A	E.F	M.A	MRSA	P.A	E.F
HHL70	*Botryosphaeria dothidea*	1	–	1	–	–	–	–	–	–	–	–	–	–	–	–	
HHL104	*Cladosporium cladosporioides*	–	–	–	–	–	–	–	–	–	–	–	–	–	–	–	–
HHL55	*Cytospora rhizophorae*	–	1	–	1	–	0.0625	0.031	–	–	1	–	1	–	1	1	–
HHL59	*Diaporthe ceratozamiae*	–	–	–	–	–	–	–	–	–	1	–	–	–	–	–	–
HHL53	*Diaporthe eucalyptorum*	–	–	–	–	–	1	–	–	1	1	–	–	–	–	–	–
HHL61	*Diaporthe perseae*	–	–	–	–	–	–	–	–	1	1	–	–	–	–	–	–
HHL7	*Diaporthe* sp.	–	–	–	–	–	1	–	–	–	–	–	–	–	–	–	–
HHL48	*Fusarium solani*	1	–	–	–	–	–	–	–	–	–	–	–	–	–	–	–
HHL96	*Guignardia mangiferae*	–	–	–	–	–	–	–	–	–	–	–	–	–	–	–	–
HHL31	*Lasiodiplodia pseudotheobromae*	–	–	–	–	–	–	–	–	–	–	–	–	–	–	–	–
HHL94	*Lasiodiplodia theobromae*	–	1	–	0.125	–	0.125	0.125	–	–	–	–	–	–	–	–	–
HHL129	*Neofusicoccum mangiferae*	–	1	–		–	–	–	–	–	0.25	–	–	–	–	–	–
HHL75	*Neofusicoccum parvum*	–	–	–	–	–	–	–	–	–	0.25	–	–	–	–	–	–
HHL46	*Neopestalotiopsis protearum*	–	–	1	–	1	0.0625	–	0.062	1	0.125	0.5	–	–	–	–	–
HHL82	*Pestalotiopsis microspora*	–	–	–	–	–	1	0.125	–	0.125	0.5	1	–	1	1	–	–
HHL51	*Pestalotiopsis palmarum*	–	–	–	–	–	–	–	–	–	–	–	–	–	–	–	–
HHL79	*Pestalotiopsis photiniae*	–		–	–	–	–	–	–	–	1	–	–	–	–	–	–
HHL10	*Pestalotiopsis* sp.	–	–	–	–	–	–	–	–	–	–	–	–	–	–	–	–
HHL56	*Pestalotiopsis theae*	–	–	–	–	–	–	–	–	–	–	–	–	–	–	–	–
HHL22	*Phomopsis asparagi*	–	–	–	–	–	–	–	–	1	–	–	–	–	–	–	–
HHL52	*Phomopsis glabrae*	–	–	–	–	–	–	–	–	–	–	–	–	–	–	–	–
HHL50	*Phomopsis longicolla*	**–**	**–**	**–**	**–**	1	1	–	–	1	0.25	–	–	–	–	–	–
HHL20	*Phomopsis *sp.	–	–	–	–	–	–	–	–	–	–	–	–	–	–	–	–
HHL38	*Seiridium ceratosporum*		–	–	–	–	–	–	–	–	0.5	–	–	–	–	–	–
HHL81	*Valsa brevispora*	–	–	–		–	–	–	–	1	0.25	–	–	–	–	–	–
Ciprofloxacin (μg/mL)		–	0.16	0.08	0.63	–	–	–	–	–	–	–	–	–	–	–	–
Amphotericin B (μg/mL)		0.04	–	–	–	–	–	–	–	–	–	–	–	–	–	–	–

^a^Strains were cultivated on four different medium, which were Dextrose Agar (PDA), Czapek’s Agar (CZA), Rice Medium (RM) and Grain Medium (GM) from *R. stylosa.*

^b^Antimicrobial activites were tested against Gram-negative [*Pseudomons adaceae* (PA)], Gram-positive [*Enterococcus faecalis* (EF), Methicillin-resistant *Staphylococcus aureus* (MRSA)] bacteria and fungi [*Monilia albican* (MA)].

**Table 2:** Antimicrobial acticity of the endophytic fungi from *R. mucronata.*

Number	Species	MIC mg/mL															
		PDA^a^				CZA				RM				GM			
		M.A^b^	MRSA	P.A	E.F	M.A	MRSA	P.A	E.F	M.A	MRSA	P.A	E.F	M.A	MRSA	P.A	E.F
HQD24	*Aspergillus fumigatus*	–	–	–	–	–	–	–	–	–	–	–	–	–	–	–	–
HQD83	*Botryosphaeria fusispora*	–	–	–	–	–	–	–	–	–	–	–	–	–	–	–	–
HQD25	*Colletotrichum gloeosporioides*	–	1	–	–	–	–	–	–	–	–	–	–	–	–	–	–
HQD62	*Diaporthe eucalyptorum*	–	–	–	–	1	–	–	1	1	–	–	–	–	–	–	–
HQD33	*Diaporthe pascoei*	1	–	–	–	–	–	–	–	–	–	–	–	–	–	–	–
HQD17	*Diaporthe phaseolorum*	–	–	–	–	–	0.062	1	1	–	–	–	–	–	–	–	–
HQD29	*Diaporthe *sp.	–	–	–	–	1	–	–	0.25	–	0.125	–	–	–	–	–	–
HQD28	*Eutypella scoparia*	–	–	–	–	–	–	–	–	–	–	–	–	–	–	–	–
HQD48	*Fusarium verticillioides*	–	–	–	0.062	–	–	0.5	–	–	–	–	–	–	–	–	–
HQD72	*Lasiodiplodia theobromae*	–	1	–	0.125	–	0.125	0.125	–	–	–	–	–	–	–	–	–
HQD23	*Neofusicoccum mangiferae*	–	1	–	–	–	–	–	–	–	0.25	–	–	–	–	–	–
HQD41	*Neofusicoccum parvum*	–	–	–	–	–	–	–	–	–	0.25	–	–	–	–	–	–
HQD5	*Neopestalotiopsis protearum*	–	–	1	–	1	0.062	–	0.062	1	0.125	0.5	–	–	–	–	–
HQD55	*Paraconiothyrium hawaiiense*	–	–	–	–	–	–	–	–	–	–	–	–	–	–	–	–
HQD20	*Pestalotiopsis microspora*	–	–	–	–	–	1	0.125	–	0.125	0.5	1	–	1	1	–	–
HQD1	*Pestalotiopsis protearum*	–	–	–	–	1	–	–	–	–	0.031	–	–	–	–	–	–
HQD6	*Pestalotiopsis *sp*.*	1	1	–	0.015	1	–	1	–	1	0.031	1	1	–	–	–	–
HQD57	*Phomopsis glabrae*	–	–	–	–	–	–	–	–	–	–	–	–	–	–	–	–
HQD8	*Phomopsis longicolla*	–	–	–	–	1	1	–	–	1	0.25	–	–	–	–	–	–
HQD47	*Pseudofusicoccum stromaticum*	–	–	–	–		–	–	–	–	–	–	–	–	–	–	–
HQD22	*Valsa brevispora*	–	–	–	–		–	–	–	1	0.25	–	–	–	–	–	–
Ciprofloxacin (μg/mL)		–	0.16	0.08	0.63	–	–	–	–	–	–	–	–	–	–	–	–
Amphotericin B (μg/mL)		0.04	–	–	–	–	–	–	–	–	–	-	–	–	–	–	–

^a^Strains were cultivated on four different medium, which were Dextrose Agar (PDA), Czapek’s Agar (CZA), Rice Medium (RM) and Grain Medium (GM) from *R. mucronata.*

^b^Antimicrobial activites were tested against Gram-negative [*Pseudomons adaceae* (PA)], Gram-positive [*Enterococcus faecalis* (EF), Methicillin-resistant *Staphylococcus aureus* (MRSA)] bacteria and fungi [*Monilia albican* (MA)].

Of the endophytic fungi isolated from *R. stylosa* (25 isolates, Table 1), RM was determined to be more suitable for antibiotic production in fungal isolates than the other three media (P < 0.05). Of these, 13 strains cultured on RM (52%) exhibited antimicrobial activity using 1 mg/mL extracts, and among them, 7 strains had stronger inhibitory effects on MRSA with MIC values less than 0.5 mg/mL. HHL55 showed the broadest antimicrobial spectrum against four indicator test microorganisms, and CZA culture of HHL55 was found to show the most potent antimicrobial activity against PA with an MIC value of 0.031 mg/mL. Only 2 strains fermented on the GM displayed low inhibitory activity with an MIC value of 1 mg/mL. In addition, three culture media derived from HHL94, HHL64 and HHL82 showed inhibitory effects on three indicator microorganisms.

Of the endophytic fungi isolated from *R. mucronata* (21 isolates, Table 2), most of the fungal strains (15 isolates, 71.4%) exhibited antimicrobial activity. A high growth inhibition rate was detected from the fungal extracts cultured on CZA (10 isolates, 47.6%) and RM (10 isolates, 47.6%) in comparison with PDA (7 isolates, 33.3%) and GM (2 isolates, 9.5%) at the selected concentration of 1 mg/mL (P < 0.05). The RM of 9 isolates and CZA of 3 isolates showed strong inhibitory effects on MRSA with MIC values less than 0.5 mg/mL. Only the extract of HQD20 cultivated on GM inhibited the growth of MRSA and MA. Moreover, HQD6 and HQD5 displayed antimicrobial activity against the growth of four indicator microorganisms tested with MIC values ranging from 0.015 to 1 mg/mL.

### Cytotoxicity of fungal extracts

**Figure 1:**
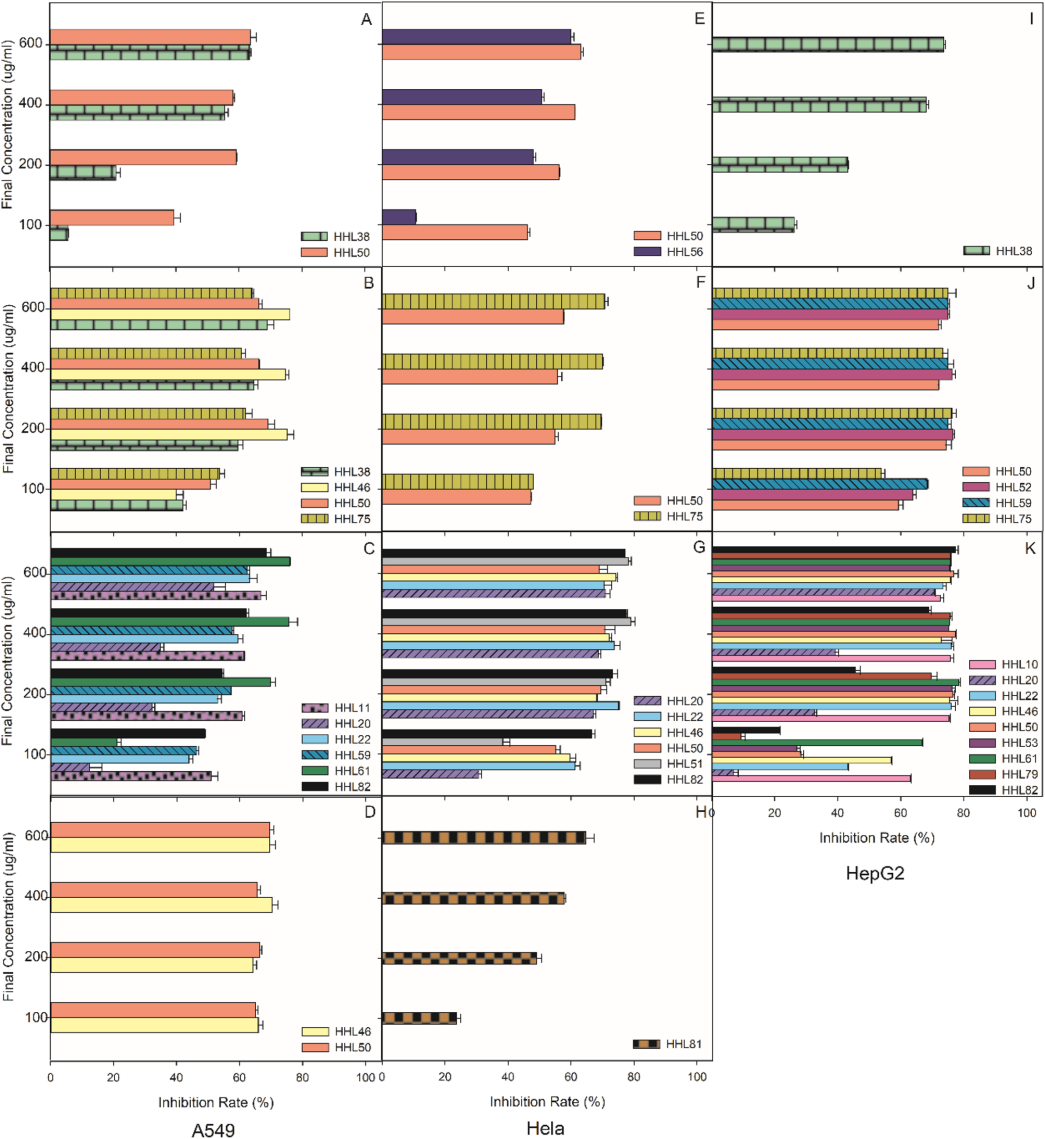
Antitumour activity of endophytic fungi from *R. stylosa*. (**A**–**D**) Shows that the fermentation products of endophytic fungi of *R. stylosa* have an inhibition rate for A549 tumour cells in different media, where (**A**) is obtained under dextrose agar (PDA) culture conditions, (**B**) is obtained under Czapek’s agar (CZA) culture conditions, (**C**) is obtained under rice medium (RM) culture conditions and (**D**) is obtained under grain medium (GM) culture conditions. (**E**–**H**) Shows that the fermentation products of endophytic fungi of *R. stylosa* have an inhibition rate for HeLa tumour cells in different media, where (**E**) is obtained under dextrose agar (PDA) culture conditions, (**F**) is obtained under Czapek’s agar (CZA) culture conditions, (**G**) is obtained under rice medium (RM) culture conditions and (**H**) is obtained under grain medium (GM) culture conditions. (**I**–**K**) shows that the fermentation products of endophytic fungi of *R. stylosa* have an inhibition rate for HepG2 tumour cells in different media, where (**I**) is results under dextrose agar (PDA) culture conditions, (**J**) is results under Czapek’s agar (CZA) culture conditions, and (**K**) is results under rice medium (RM) culture conditions.

**Figure 2:**
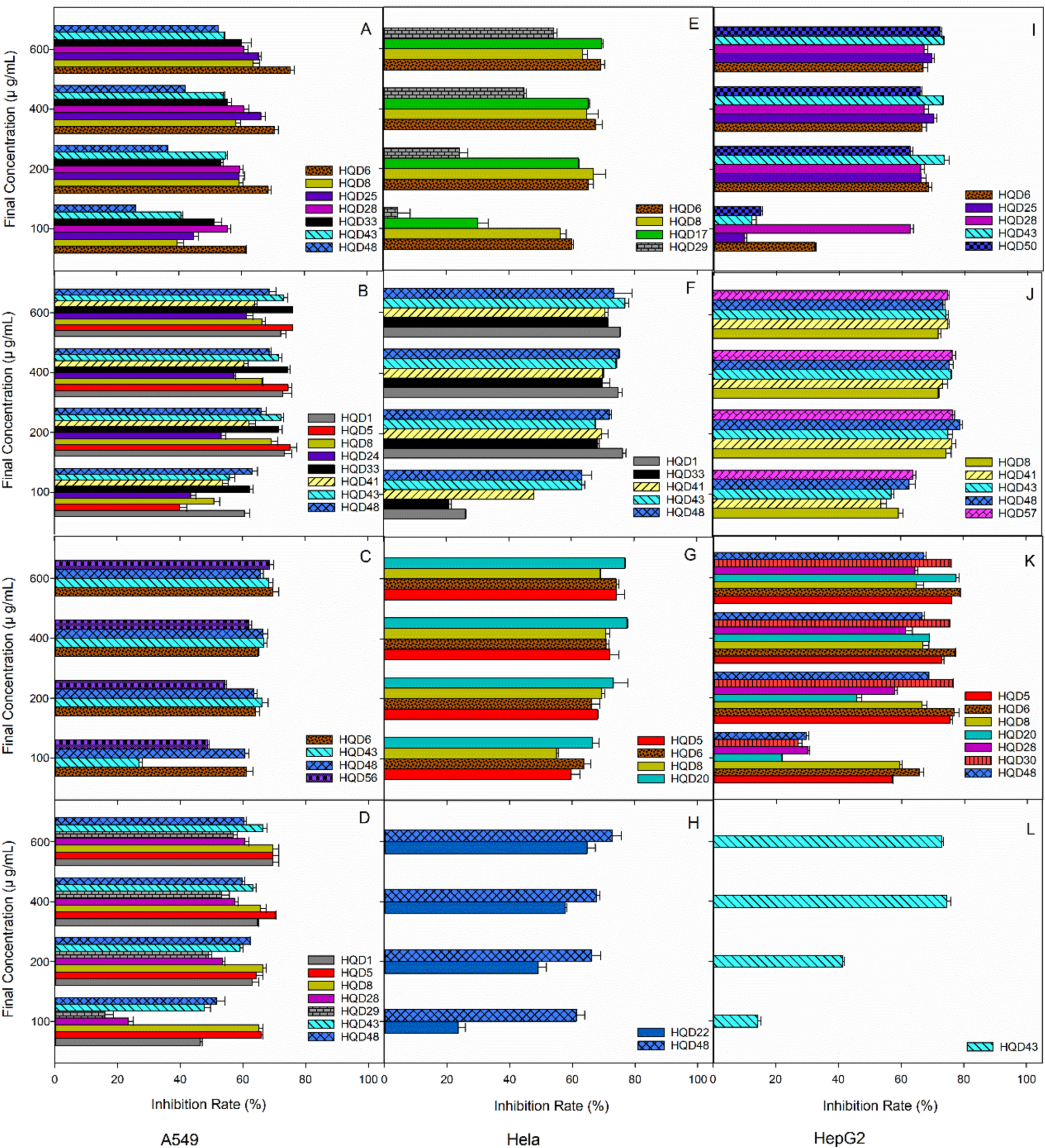
Antitumour activity of endophytic fungi from *R. mucronata*. (**A**–**D**) Shows that the fermentation products of endophytic fungi of *R. mucronata* have an inhibition rate for A549 tumor cells in different media, where (**A**) is obtained under dextrose agar (PDA) culture conditions, (**B**) is obtained under Czapek’s agar (CZA) culture conditions, (**C**) is obtained under rice medium (RM) culture conditions and (**D**) is obtained under grain medium (GM) culture conditions. (**E**–**H**) Shows that the fermentation products of endophytic fungi of *R. mucronata* have an inhibition rate for HeLa tumor cells in different media, where (**E**) is obtained under dextrose agar (PDA) culture conditions, (**F**) is obtained under Czapek’s agar (CZA) culture conditions, (**G**) is obtained under rice medium (RM) culture conditions and (**H**) is obtained under grain medium (GM) culture conditions. (**I**–**L**) Shows that the fermentation products of endophytic fungi of *R. mucronata* have an inhibition rate for HepG2 tumor cells in different media, where (**I**) is results under Dextrose Agar (PDA) cultural condition, (**J**) is results under Czapek’s Agar (CZA) cultural condition, (**K**) is results under Rice Medium (RM) cultural condition, and (**L**) is results under Grain Medium (GM) cultural condition.

The cytotoxic effects of *R. stylosa* and *R. mucronata* endophytic fungal extracts were tested against HeLa, A549 and HepG2 cells using an MTT colorimetric assay. The half inhibitory concentration (IC_50_) values are shown in Tables 3 and 4. The results are presented as the means ± standard deviation of experiments performed in triplicate. Doxorubicin was used as the positive control, and the IC_50_ values were 0.17 ± 0.01 nM, 8.6 ± 0.1 nM and 137 ± 0.9 nM for A549, HeLa and HepG2 cells, respectively, which were stronger than the cytotoxicity of the fungal extracts. Cell viability using different concentrations of these extracts after 24 h of treatment was determined (Figs. 1 and 2). The cytotoxicity of fungal extracts showed a dose-dependent relationship.

**Table 3:** Anti-tumor activity of endophytic fungi from *R. stylosa.*

NO	Species	IC_50_ μg/mL											
		﷐A549				Hela				HepG2			
		PDA	CZA	RM	GM	PDA	CZA	RM	GM	PDA	CZA	RM	GM
HHL70	*Botryosphaeria dothidea*	–	–	–	–	–	–	–	–	–	–	–	–
HHL104	*Cladosporium cladosporioides*	–	–	–	–	–	–	–	–		–	–	–
HHL55	*Cytospora rhizophorae*	–	–	–	–	–	–	–	–	–	–	–	–
HHL59	*Diaporthe ceratozamiae*	–	–	140.46 ± 5.61	–	–	–	–	–	–	26.54 ± 3.73	–	–
HHL53	*Diaporthe eucalyptorum*	–	–	–	–	–	–	–	–	–	–	185.03 ± 3.22	–
HHL61	*Diaporthe perseae*	–	–	201.04 ± 1.22	–	–	–	–	–	–	–	23.17 ± 4.26	–
HHL7	*Diaporthe *sp*.*	–	–	–	–	–	–	–	–	–	–	–	–
HHL48	*Fusarium solani*	–	–	–	–	–	–	–	–	–	–	–	–
HHL96	*Guignardia mangiferae*	–	–	–	–	–	–	–	–	–	–	–	–
HHL31	*Lasiodiplodia pseudotheobromae*	–	–	–	–	–	–	–	–	–	–	–	–
HHL94	*Lasiodiplodia theobromae*	–	–	–	–	–	–	–	–	–	–	–	–
HHL129	*Neofusicoccum mangiferae*	–	–	–	–	–	–	–	–	–	–	–	–
HHL75	*Neofusicoccum parvum*	–	64.38 ± 3.40	–	–	–	109.77 ± 1.21	–	–	–	84.23 ± 1.61	–	–
HHL46	*Neopestalotiopsis protearum*	–	144.39 ± 1.58	–	11.65 ± 0.34	–	–	31.03 ± 1.21	–	–	–	69.15 ± 1.63	–
HHL82	*Pestalotiopsis microspora*	–	–	123.35 ± 1.29	–	–	–	14.38 ± 1.84	–	–	–	237.47 ± 1.75	–
HHL51	*Pestalotiopsis palmarum*	–	–	-	–	–	–	142.77 ± 1.97	–	–	–	–	–
HHL79	*Pestalotiopsis photiniae*	–	–	-	–	–	–	-	–	–	–	224.42 ± 1.43	-
HHL10	*Pestalotiopsis *sp*.*	–	–	97.21 ± 1.36	–	–	–	-	–	–	–	39.09 ± 1.38	-
HHL56	*Pestalotiopsis theae*	–	–	-	–	–	–	-	–	–	–	-	-
HHL22	*Phomopsis asparagi*	–	–	170.84 ± 1.99	–	341.14 ± 1.37	-	-	–	–	–	137.13 ± 1.71	-
HHL52	*Phomopsis glabrae*	–	–	–	–	–	–	70.55 ± 1.37	–	–	33.04 ± 1.21	-	-
HHL50	*Phomopsis longicolla*	192.96 ± 2.62	108.53 ± 2.09	–	16.31 ± 0.36	137.73 ± 2.53	120.38 ± 1.79	73.18 ± 1.64	–	–	55.05 ± 1.92	177.71 ± 4.76	-
HHL20	*Phomopsis *sp*.*	–	–	605.5 ± 1.51	–	–	–	190.74 ± 1.39	–	–	–	378.08 ± 1.59	-
HHL38	*Seiridium ceratosporum*	386.35 ± 3.82	151.1 ± 2.25	–	–	–	–	–	–	239.82 ± 1.89	–	–	–
HHL81	*Valsa brevispora*	–	–	–	–	–	–	–	277.67 ± 2.02	–	–	–	–
Doxirubicin(nM)		0.17 ± 0.01				8.6 ± 0.1				137 ± 0.9

**Table 4:** Anti-tumor activity of endophytic fungi from *R. mucronata.*

NO	Species	IC_50_μg/mL											
		A549				Hela				HepG2			
		PDA	CZA	RM	GM	PDA	CZA	RM	GM	PDA	CZA	RM	GM
HQD24	*Aspergillus fumigatus*	–	179.67 ± 1.21	–	–	–	–	–	–	–	–	–	–
HQD83	*Botryosphaeria fusispora*	248.17 ± 1.22	62.63 ± 1.63	208.13 ± 1.42	112.17 ± 1.63	–	30.62 ± 1.21	–	–	223.05 ± 1.51	67.73 ± 1.22	–	262.05 ± 1.45
HQD25	*Colletotrichum gloeosporioides*	144.89 ± 1.71	–	–	–	–	–	–	–	241.22 ± 6.19	–	–	–
HQD62	*Diaporthe eucalyptorum*	–	–	–	–	–	–	–	–	–	–	182.03 ± 1.32	–
HQD33	*Diaporthe pascoei*	109.64 ± 1.25	23.62 ± 1.96	–	–	–	214.44 ± 1.57	–	–	–	–	–	–
HQD17	*Diaporthe phaseolorum*	–	–	–	–	201.66 ± 1.12	–	–	–	–	–	–	–
HQD29	*Diaporthe *sp*.*	–	–	–	331.91 ± 1.33	498.96 ± 1.66	–	–	–	–	–	–	–
HQD28	*Eutypella scoparia*	53.81 ± 1.37	–	–	277.58 ± 1.30	–	–	–	–	14.75 ± 1.21	–	227.03 ± 1.26	–
HQD48	*Fusarium verticillioides*	547.42 ± 1.65	4.83 ± 1.61	34.57 ± 1.25	106.79 ± 1.33	–	66.78 ± 1.72	–	19.83 ± 1.13	237.56 ± 1.2	60.8 ± 1.29	201.75 ± 1.47	–
HQD72	*Lasiodiplodia theobromae*	–	–	–	–	–	–	–	–	–	–	–	–
HQD23	*Neofusicoccum mangiferae*	–	–	–	–	–	–	–	–	–	–	–	–
HQD41	*Neofusicoccum parvum*	–	64.38 ± 1.23	–	–	–	109.77 ± 1.22	–	–	–	84.23 ± 1.63	–	–
HQD5	*Neopestalotiopsis protearum*	–	144.40 ± 1.75	–	11.65 ± 1.03	–	–	31.03 ± 1.21	–	–	–	69.15 ± 1.63	–
HQD55	*Paraconiothyrium hawaiiense*	–	–	–	–	–	–	–	–	–	–	–	–
HQD20	*Pestalotiopsis microspora*	–	–	126.73 ± 1.21	–	–	–	14.38 ± 1.84	–	–	–	237.47 ± 1.75	
HQD1	*Pestalotiopsis protearum*	–	40.09 ± 1.39	–	119.65 ± 1.38	–	186.85 ± 1.67	–	–	–	–	–	–
HQD6	*Pestalotiopsis *sp.	28.90 ± 1.26	–	14.99 ± 1.62	–	–	–	–	–	196.17 ± 1.65	–	9.58 ± 0.01	–
HQD57	*Phomopsis glabrae*	–	–	–	–	–	–	–	–	–	33.04 ± 1.21	–	–
HQD8	*Phomopsis longicolla*	192.97 ± 1.86	108.53 ± 2.21	–	16.31 ± 1.26	–	–	–	–	–	–	–	–
HQD47	*Pseudofusicoccum stromaticum*	–	–	–	–	–	–	–	–	–	–	–	–
HQD22	*Valsa brevispora*	–	–	–	–	–	–	–	277.67 ± 1.21	–	–	–	–
Doxirubicin (nM)		0.17 ± 0.01				8.6 ± 0.1				137 ± 0.9

As shown in Table 3, of the endophytic fungi isolated from *R. stylosa*, extracts of 10 isolates (40%) tested were cytotoxic and exhibited a viability percentage of A549 cells ≤ 50%, with all of the *Phomopsis *sp. and 3 isolates (50% of the *Pestalotiopsis *sp.) showing antitumour activity, and the IC_50_ values of four extracts (HHL75, HHL46, HHL10 and HHL50) were lower than 100 μg/mL (IC_50_ values of 64.38 ± 3.40, 11.65 ± 0.34, 97.21 ± 1.36 and 16.31 ± 0.36 μg/mL, respectively), suggesting the cytotoxic potential of these four fungal isolates; 75% of them belong to *Phomopsis *sp*.* and *Pestalotiopsis *sp*.* (Fig. 1A–D). Extracts of 9 isolates (36%) showed cytotoxicity and displayed a HeLa cell viability percentage ≤ 50%. In particular, the HHL46, HHL82, HHL52 and HHL50 extracts showed the most significant cytotoxic effect, with IC_50_ values of 31.03 ± 1.21, 14.38 ± 1.84, 70.55 ± 1.37 and 73.18 ± 1.64, respectively, which were lower than 100 μg/mL. The four fermentation products that inhibited HeLa cells were all from *Phomopsis *sp*.* and *Pestalotiopsis* sp., which coincided with the inhibition of A549 cells (Fig. 1E–H). Extracts of 13 isolates (52%) showed cytotoxicity against HepG2 cells, among which 7 extracts (HHL61, HHL75, HHL46, HHL10, HHL52 and HHL50) could significantly suppress the proliferation of HepG2 cells with IC_50_ values below 100 μg/mL. Eight of the 13 isolates (61.54%) belong to *Phomopsis *sp. and *Pestalotiopsis* sp. (Fig. 1I–K). *Phomopsis *sp. and *Pestalotiopsis *sp. were the dominant fungi (41.67%) in *R. stylosa*, with 9 strains inhibiting at least one tumour cell, suggesting that these two fungal genera could have high potential as producers of antitumour compounds.

As shown in Table 4, of the endophytic fungi isolated from *R. mucronata*, extracts of 13 isolates (61.9%) displayed cytotoxic activity on A549 cells, 10 isolates (47.6%) on HeLa cells, and 10 isolates (47.6%) on HepG2 cells. Of these, 9 extracts (HQD83, HQD33, HQD28, HQD48, HQD41, HQD5, HQD1, HQD6 and HQD8) exhibited significant cytotoxicity against A549 cells with IC_50_ values less than 100 μg/mL (Fig. 2A–D). Four extracts (HQD83, HQD48, HQD5 and HQD20) showed strong cytotoxic effects against the HeLa cell line with IC_50_ values below 100 μg/mL (Fig. 2E–H). Seven extracts (HQD83, HQD28, HQD48, HQD41, HQD5, HQD6 and HQD57) were potent (IC_50_ < 100 μg/mL) against HepG2 cells (Fig. 2I–L). *Diaporthe *sp. and *Pestalotiopsis *sp. were dominant fungi (38.09%) in *R. mucronata*, with all of them displaying at least one tumour cell. However, the three isolates with the strongest ability to inhibit A549, HeLa and HepG2 cells were *Fusarium verticillioides*, *Pestalotiopsis microspore* and *Eutypella scoparia*, respectively, and none of them were *Diaporthe* sp*.*

In an attempt to promote antitumour substance production, four different media were adopted for fungal isolate cultivation to activate biosynthetic silencing of gene expression. We found that extracts of 1 isolate (HQD28) cultured on PDA, 1 isolate (HQD48) cultured on CZA, 3 isolates (HHL82, HQD52 and HQD6) cultured on RM and 4 isolates (HHL46, HQD48, HQD5 and HQD8) cultured on GM exhibited significant antiproliferative activity against at least one of the tested carcinoma cells with an IC_50_ < 20 μg/mL. GM culture of *R. stylosa* endophytic HHL46 and RM culture of HHL61 and HHL82 were most effective against A549, HeLa and HepG2 cells, with IC_50_ values of 14.38 ± 1.84 μg/mL, 23.17 ± 4.26 μg/mL and 14.38 ± 1.84 μg/mL, respectively. The significant difference analysis showed that the inhibition of HeLa cells by the products of endophytic fungi of *R. stylosa* cultured on RM was stronger than those cultured under the other three conditions (P < 0.05). No significant difference (P > 0.05) was observed between the cytotoxicity for A549 of samples from four cultural media having equal degrees at end of 24 h (Table 3). The extracts from CZA culture of *R. mucronata* endophytic HQD48 and RM culture of HQD20 and HQD6 exhibited cytotoxicity towards A549, HeLa and HepG2 cells, with IC_50_ values of 4.83 ± 1.61 μg/mL, 14.38 ± 1.84 μg/mL and 9.58 ± 0.01 μg/mL, respectively (Table 4). The genera *Pestalotiopsis* and *Phomopsis* were demonstrated to be rich sources of antitumour secondary metabolites. Notably, RM culture of HQD6 exhibited cytotoxic and antiproliferative effects against three tested cancer cell lines with IC_50_ values ranging from 9.58 to 14.99 μg/mL.

### Profiling of bioactive metabolites by HPLC

**Figure 3:**
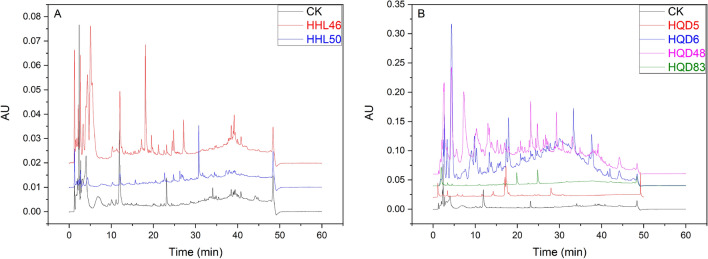
HPLC chromatogram fermented endophytic fungi produced in rice medium (RM) fermentation. (**A**) Shows the chromatograms of fermented product grown on the RM at 254 nm for 2 endophytic fungi of *R. stylosa* (HHL46 and HHL50) with strong bioactive abilities and the extraction of RM as control. (**B**) Shows the chromatograms of fermented product grown on the RM at 254 nm for 4 endophytic fungi of *R. mucronata* (HQD5, HQD6, HQD48 and HQD83) with strong bioactive abilities and the extraction of RM as control. Among them CK means, control check, the result of HPLC from the extraction of rice medium.

According to the screening for antimicrobial activity and cytotoxicity, we found that 6 endophytic fungi showed strong bioactive abilities: HHL46, HHL50, HQD5, HQD6, HQD83 and HQD48. The difference significance analysis showed that RM was to be the best to produce active metabolites and the product diversity of endophytic fungi on RM was analyzed by HPLC in further. Figure 3 shows the chromatograms of the fermented products of these endophytic fungi at 254 nm. The HPLC analysis results provide a wealth of information, and the hydrophilic compounds were found in the first 10 min. Within 10 to 15 min, the moderately polar components and hydrophobic components were washed out. RM cultures of HQD5 and HQD6 showed more diverse secondary metabolites than other cultures.

## Discussion

Infectious diseases, including bacterial infections, pose a serious threat to global health and drug resistance^23,24^. Unfortunately, there has been resistance detected against every antibiotic on the market, and if antibiotic resistance is not mitigated, pathogenic bacteria will once again become one of the leading causes of mortality, with an estimated yearly death toll of ~ 10 million by 2050^25^. The development of new antimicrobial agents is pointed out as an effective solution to address this problem^26,27^. As a promising source of diverse and structurally unprecedented bioactive natural products, mangrove-derived endophytic fungi are unquestionably important and continuously attract considerable attention^28,29^. In our current study, of 46 endophytic fungal strains investigated, 32 extracts (69.6%) exhibited antimicrobial activity, which is consistent with the findings of a previous study by Buatong et al*.*^30^ in which 61.3% of mangrove fungal endophytes produced inhibitory compounds.

Cancer-related death is one of most significant threats to human health worldwide, with an estimated 12.7 million new cases and 7.6 million cancer deaths each year^31^. At present, the primary treatment method for cancers is to combine removing the tumour with anaesthetic agents after surgery^32^. Unfortunately, the surgical process possibly leads to tumour progression, causing a large number of tumour cells to be released and reducing the activity of T, B and NK lymphocytes in the postoperative period^33^. It has also generated a large body of information that is being harnessed to develop new therapeutic modalities for treating cancer^34^; however, the search for cytotoxic agents that selectively impact proliferating cells still plays an essential role in tumour treatment^35^. In our study, of 46 endophytic fungal strains investigated, 23 extracts (50%) showed cytotoxicity, 21 extracts (46.65%) were cytotoxic against A549 cells, 16 extracts (34.78%) were cytotoxic against HeLa cells, and 21 extracts (46.65%) were cytotoxic against HepG cells. This was in accordance with previous reports, in which 9 endophytic fungi were successfully obtained from the leaves of *Ginkgo biloba*. The extracts of isolates J-1, J-2 and J-3 markedly inhibited the proliferation of HeLa cells, promoted their apoptosis and blocked their migration^36^, and 12 isolate extracts (85.7%) derived from mangrove *Rhizophora mucronata* were cytotoxic (cell viability < 50%) against T47D cells^12^. In our current study, we found that *Pestalotiopsis *sp*.* HQD6 displayed significant antitumour activity with IC_50_ values below 20 μg/mL and showed more diverse secondary metabolites than other formulations, which was in accordance with our previous reports that demethylincisterol A_3_ was isolated from the *R. mucronata* endophytic *Pestalotiopsis *sp. HQD6 and showed significant in vitro cytotoxicity against the human cancer cell lines HeLa, A549 and HepG, with IC_50_ values reaching nM ranging from 0.17 to 14.16 nM^19^. It also reported to be a selective inhibitor of the classical nonreceptor protein tyrosine phosphatase Shp2^20^.

Culture-dependent methods have been developed aiming at substantial increases in biologically active secondary metabolite production by any given microorganism^37^. RM was demonstrated to have the highest suitability for antibiotic production; 23 extracts (50%) showed antimicrobial activity against at least one of four strains, and the extract of HQD1 exhibited antimicrobial activity against MRSA with an MIC value of 0.031 mg/mL, which was in accordance with the previous reports of Rivai et al.^12^. RM (11 isolates, 39.13%) and GM (9 isolates, 19.56%) were more suitable for antitumour agent production, which agreed with previous reports of GM-cultivated *Oidiodendron truncatum* leading to the discovery of the potent anticancer agent chetracin B with cytotoxicity against five human cancer lines reaching nM degree^38^. The variation in the antibiotic and cytotoxic properties among media could possibly be related to the composition of RM, and GM activated our isolated fungal biosynthetic gene clusters^39^.

## Materials and methods

### Fungal material used

Previously isolated endophytic fungi from healthy roots, stems, leaves, hypocotyls and flowers of *R. stylosa* (No. 201510-HHL) and *R. mucronata* (No. 201510-HQD) were used for the present study. The titled plant materials were collected from a specific location (110°32′–110°37′ E, 19°51′–20°01′ N) under the permission of Dong Zhai Gang Mangrove National Nature Reserve Authority of Hainan Island, China. Based on the consideration on the conservation of mangrove plants resources, we had to collect only a few individuals per species. The study’s authors promise the use of plants in the present study complies with international, national and/or institutional guidelines. These endophytic fungi were identified in combination with morphologic characteristics and p internal transcribed spacer (ITS) sequences. Fungal ITS-rDNA sequences of 46 representative isolates were deposited in GenBank under the accession number KX618209 and numbers ranging from KX631698 to KX631742, and their antioxidant capacity has also been demonstrated^22^.

### Fermentation and extraction

Fungal isolates were cultured on Petri dishes of potato dextrose agar (PDA) at 28 °C for 5 days and then inoculated on four different media, PDA, Czapek’s agar (CZA), rice medium (RM), and grain medium (GM), for stationary fermentation. After 40 days, the harvested cultures were extracted with 100 mL of ethyl acetate, ultrasonicated at 50 °C for 1 h, and then filtered through filter paper. A rotary evaporator was used at 50 °C with low pressure to evaporate the remaining ethyl acetate. The extracts were dissolved in dimethyl sulfoxide (DMSO, not exceeding 1%, v/v) and stored at 4 °C until use.

### Screening for antimicrobial activity

The endophytic fungal extracts were tested against the gram-negative *bacterium Pseudomonas adaceae* (PA), gram-positive bacteria *Enterococcus faecalis* (EF), methicillin-resistant *Staphylococcus aureus* (MRSA) and pathogenic fungus *Monilia albicans* (MA) by the microdilution method with some modifications^40^. An aliquot of extract (2 μL) was added to 198 μL of the indicator strain suspension at a density of 5 × 10^6^ CFU/mL into each well of a 96-well microplate. This mixture was then incubated for 24 h/48 h at 37 °C/28 °C, and the minimum inhibitory concentration (MIC) was recorded. Ciprofloxacin was used as a positive control for bacteria, and amphotericin B was used as a positive control for fungi.

### Screening for cytotoxicity

Cytotoxicity was tested by the 3-(4,5-dimethylthiazol-2-yl)-2,5-diphenyltetrazolium bromide (MTT) method according to our previous report^20^. Human cervical carcinoma cells (HeLa), human lung cancer cells (A549), and human hepatoma cells (HepG2) were purchased from Cell Resource Center, Shanghai Institutes for Biological Sciences, Chinese Academy of Sciences and the cell lines were grown in RPMI-1640 culture medium with 200 μL/mL foetal bovine serum (FBS) under a humidified atmosphere of 5% CO_2_ and 95% air at 37 °C. A 100 μL cell suspension at a density of 1.5 × 10^5^ cell mL^−1^ was pipetted into 96-well microtiter plates. Fungal extracts with different concentrations from 100 to 600 μg/mL were added to each well and incubated for 24 h under the above conditions in a CO_2_ incubator. Then, 20 μL of MTT (5 mg/mL) was added to each well, and the plates were further incubated for 3 h. DMSO (200 μL) was added to dissolve the formazan crystals. The absorbance was then measured at 570 nm by a microplate reader. The cell inhibition rate (%IR) was calculated by the following equation: % IR = [(*A*_*bla*_ − *A*_*sam*_)/*A*_*bla*_] × 100, where *A*_*bla*_ is the absorbance of the blank and *A*_*sam*_ is the absorbance of the test compounds. The IC_50_ value was calculated from the dose–response relationship. Doxorubicin was used as the positive control.

### The profiling of secondary metabolites by HPLC

To assign the bioactivities of the endophytic fungal cultural extract, we performed profiling of the compounds with a Waters 2998 series HPLC system. Separation was achieved using a 250 mm column at 26 °C with a multistep linear gradient elution program in which chromatographic methanol changed from 0 to 20% in 5 min, from 20 to 25% in 5–15 min, from 25 to 50% in 15–30 min, from 50 to 65% in 30–40 min, from 65 to 85% in 40–5 50 min, and finally from 85 to 100% in 50–60 min. UV spectra were recorded at 254 nm. The extraction of rice medium was used as a control^41^.

### Statistical analyses

SPSS 23.0 software was used for statistical analysis, Ordinary one-way ANOVA and principal component analysis PCA used for the different measured variables in the study. Significance was evaluated in at a level of P < 0.05, for the endophyte extract of different medium to antimicrobial (PCA) and cytotoxic activities (ANOVA) were performed using Tim Duncan’s test. All errors are expressed as standard deviations (SD).

## Conclusion

To date, there are few systematic studies on the antimicrobial and antitumour potential of mangrove endophytic fungi. Our study indicated that the antimicrobial and cytotoxic activities of mangrove endophytic fungal extracts grown on four media showed distinguishable differences in activities and revealed that RM could promote the secretion of bioactive secondary metabolites. *Neopestalotiopsis protearum* HQD5 and *Pestalotiopsis *sp*.* HQD6 showed potent antimicrobial activity; *Neopestalotiopsis protearum* HHL46, *Phomopsis longicolla* HHL50, *Botryosphaeria fusispora* HQD83, *Fusarium verticillioides* HQD48 and *Pestalotiopsis *sp*.* HQD6 displayed significant antitumour activity. Considering these results, these fungi could be further explored for the characterization of antimicrobial and cytotoxic secondary metabolites, which could explain the significant biological activities of the abovementioned fungal strain.
